# 

**Published:** 1891-02

**Authors:** 


### BOOKS AND PAMPHLETS RECEIVED.

Text-Book of Hygiene. A Comprehensive Treatise on the Principles and Practice of Preventive Medicine from an American Standpoint. By George H. Rohé, M. D., Professor of Obstetrics in the College of Physicians and Surgeons, Baltimore ; Member of the American Public Health Association ; foreign Associate of the Société Française d' Hygiéne, etc. Second edition, thoroughly revised and largely re-written, with many illustrations and valuable tables. 8vo, pp.x. ---421. Philadelphia and London : F. A. Davis. 1890. Price, $2.50 net.

Twelve Lessons on the Structure of the Central Nervous Systems for Physicians and Students. By Dr. Ludwig Edinger, Frankfort-on-the-Main. Second revised edition, with 133 illustrations. Translated by Willis Hall Vittum, M. D., St. Paul, Minn. Edited by C. Eugene Riggs,

A. M., M. D., Professor of Mental and Nervous Diseases, University of Minnesota; Member of the American Neurological Association. 8vo, pp. xii.—230. Philadelphia and London: F. A. Davis. 1890. Price, $1.75 net.

An Illustrated Encyclopedic Medical Dictionary. Being a dictionary of the technical terms used by writers on medicine and the collateral sciences, in the Latin, English, French and German languages. By Frank P. Foster, M. D., editor of the *New York Medical Journal*, assisted by eleven collaborators. Vol. II. Cac—Fasay. With illustrations. 4to, pp. 792. New York: D. Appleton & Co. 1890.

Professor Koch's Method to Cure Tuberculosis Popularly Treated. By Dr. Max Birnbaum. Translated from the German by Dr. Fr. Brendecke. With an appendix, being Prof. Koch's first communication on the subject, translated from the Deutsche Medicinische Wochenschrift, and Explanatory Notes by the Author. Milwaukee, Wis.: H. E. Haferkorn. 1891. Price, paper, 75 cents; cloth, $1.00.

A Text-Book of the Diseases of the Ear. By Dr. Josef Gruber, Professor of Otology in the Imperial Royal University of Vienna, etc. Translated from the Second German edition by special permission of the author, and edited by Edward Law, M. D., C. M., Edin., M. R. C. S., Eng., Surgeon to the London Throat Hospital for Diseases of the Throat, Nose and Ear; and by Coleman Jewell, Lond., M. D., M. R. C. S., Eng. Late Physician and Pathologist to the London Throat Hospital. With 150 illustrations and 70 colored figures on two lithographic plates. Cloth, large 8vo, pp. xxiv.–580. New York. D. Appleton & Co. 1890. Buffalo: Otto Ulbrich.

The Surgical Conception of Peritonitis. By Joseph Price, M. D., Philadelphia, Pa. Reprint, Vol. III., Transactions American Association of Obstetricians and Gynecologists. 1890.

A Large Group of Mixed Specimens. Illustrating the Principal Complications and Varieties of Pelvic and Abdominal Surgery. By Joseph Price, M. D., Philadelphia, Pa. Reprint, Vol. III., Transactions American Association of Obstetricians and Gynecologists. 1890.

Certain Causes of Major Pelvic Troubles Traceable to Minor Gynecology. By Joseph Price, M. D., Philadelphia, Pa. Reprint, Transactions Philadelphia County Society. 1890.

Timely Topics for Medical Men. Koch's Discovery (Illustrated). Alimentation as a Therapeutic Measure. The Arlington Chemical Co., Yonkers, N. Y.

Census Bulletin No. 16, December 12, 1890. Population of the United States by States and Territories. 1890. By Robert P. Porter, Superintendent of the Eleventh Census.

Third Annual Report of the Methodist Episcopal Hospital, Brooklyn,

N. Y., covering the period from November 1, 1889, to October 31, 1890. Through Lewis S. Pilcher, M. D., one of the attending surgeons.

Annual Report of the Rhode Island Hospital. 1890. Providence, R. I: E. L. Freeman & Son, Printers to the State.

Twentieth Annual Report of the Managers of the Buffalo State Hospital, for the year 1890. Albany: James B. Lyon, State Printer. 1891.

Bulletin of the American Academy of Medicine. No. 1. January, 1891.

Bulletin of the Agricultural Experiment Station. Botanical Division, Cornell University, College of Agriculture, xxiv.; December, 1890. The Clover Rust. Ithaca: Published by the University. 1890.

The Atmospheric Tractor. A New Instrument, and Some New Theories in Obstetrics. By Peter McCahey, M. D., Philadelphia. Reprint from *Medical and Surgical Reporter*, November 29, 1890.

Recent Therapeutical Notes on the Use of Papoid (Carica Papaya) in the Treatment of Dyspepsia and Diphtheria. Johnson & Johnson, Manufacturing Chemists, 92 William street, New York. 1890.

Bulletin of the Experiment State Entomological Division. Cornell University, Department of Agriculture, xxiii.; December, 1890. Insects Injurious to Fruits. Ithaca: Published by the University. 1890.

Internal Oesophogotomy. Report of a Case. By John O. Roe, M. D., Rochester, N. Y. Reprint, BUFFALO MEDICAL AND SURGICAL JOURNAL, May, 1890.

Instruments Presented at the Tenth and Eleventh Meetings of the American Laryngological Association. By John O. Roe, M. D., Rochester, N. Y. Reprint.

The Treatment of Diseased Tonsils when unattended with Hypertrophy. By John O. Roe, M. D., Rochester, N. Y. Reprint: *New York Medical Journal*, October 26, 1889.

Pyoktanin in Diseases of the Eye, Ear and Throat. By W. Cheatham, M. D., Louisville, Ky. Reprint: *The Lancet-Clinic*, November 15, 1890.

Removal of Tonsillar Hypertrophy by Electro-Cautery Dissection. By Edwin Pynchon, M. D. Reprint: *Journal of the American Medical Association*, November 22, 1890.

Fifty Remembers for Druggists. By H. M. Whelpley, M. D., Ph. G., St. Louis, Mo.

Abnormal Intra-Thoracic Air Pressures and Their Treatment. Address at the Seventh Annual Meeting of the American Climatological Association, September 2, 1890. By Charles Denison, A. M., M. D., Denver, Colo., President. Reprint: *The Sanitarian*, November, 1890.

Hydrophobia. By Hiram Corson, M. D., Conshohocken, Pa. Reprint: *Medical and Surgical Reporter*, November 29, 1890.

Note on the Virile Reflex. By C. H. Hughes, M. D., St. Louis, Mo. Reprint: From *Alienist and Neurologist*, January, 1891.

The Psycopathic Sequences of Hereditary Enlargement. By C. H. Hughes, M. D., St. Louis, Mo. Reprint: *The Alienist and Neurologiist*, October, 1890.

Medical Department, University of Wooster. Announcement for 1891. Cleveland, O.

HEALTH BULLETINS.

Michigan State Board of Health. Health in Michigan, December, 1890.

Summary of Deaths in Newport, R. I., for the year 1890.

Newport, R. I.: Report of Deaths and Contagious Diseases for December, 1890.

I. Pennsylvania State Board of Health. Circular No. 29. The Dangers Arising from Public Funerals of those who have Died from Contagious and Infectious Diseases. Addressed to the Clerical Profession.

II. Circular No. 30. The Disposal of the Sewage of Public Edifices. Addressed to the Managers of Public Institutes.

III. Circular No. 31. Precautions to be Adopted by Funeral Directors to Prevent the Spread of Contagious and Infectious Diseases.

Abstracts of Sanitary Reports U. S. Marine Hospital Service. Volume VI., Numbers 1, 2, 3, and 4.

Notice to Contributors. — We are glad to receive contributions from every one who knows anything of interest to the profession. Articles designed for publication in the Journal should be handed in before the first day of the month. The Editors are not responsible for the views or opinions of contributors. All communications should be addressed to

284 Franklin Street, Buffalo, N. Y.

